# Supplementary material for: Heterobilayers of 2D materials as a platform for excitonic superfluidity
Source: Nat Commun. 2020 Jun 12;11:2989. doi: 10.1038/s41467-020-16737-0 (PMC7293212; doi:10.1038/s41467-020-16737-0)
Supplement: Supplementary file 1 — Supplementary Information [file 41467_2020_16737_MOESM1_ESM.pdf]

# Supplementary Information for: “Heterobilayers of 2D materials as a platform for excitonic superfluidity”

Sunny Gupta[1], Alex Kutana[1], and Boris I. Yakobson[1]

[1]Materials Science and NanoEngineering, Rice University, Houston, TX 77005, USA

## Supplementary Note 1

### 1.1 Model Hamiltonian analysis - exciton binding energy

The model Hamiltonian within the effective mass approximation for an electron-hole pair in a bilayer semimetal is given by

$$H = -\frac{\hbar^2}{2\mu_x}\frac{\partial^2}{\partial x^2} - \frac{\hbar^2}{2\mu_y}\frac{\partial^2}{\partial y^2} + V_{\mathrm{eh}}\left(r\right) \tag{1}$$

Here, $\mu_{x,y}$ are the reduced masses in $x$ and $y$ directions, respectively, and $V_{\mathrm{eh}}\left(r\right)$ is the effective 2D Coulomb potential in a bilayer metal. Unlike 2D single-layer [1] and bilayer [2] semiconductors, where intralayer and interlayer exciton binding energies are much larger compared to bulk due to weak screening, in semimetallic systems interactions are reduced significantly, leading to weaker binding. Here we adopt the random phase approximation (RPA) form of the screened potential suitable for metallic bilayers [3, 4], given by

$$V_{\mathrm{eh}}\left(q\right) = \frac{2\pi q}{\kappa}\frac{e^{-qd}}{\left(q+s\right)^2 - s^2 e^{-2qd}} \tag{2}$$

Here, $\kappa$ is the background dielectric constant, $s$ is the 2D screening wave number and $d$ is the distance between the layers. In the ideal case of a two-band metal, the dependence of the 2D screening parameter $s$ on temperature and carrier concentration has been adapted from Ref. [5] and is given by

$$s = \frac{1}{2}\left[g_{\mathrm{h}}\sqrt{m^*_{\mathrm{h}x}m^*_{\mathrm{h}y}}\left(1 - e^{-\frac{2\pi n}{g_{\mathrm{h}}k_{\mathrm{B}}T\sqrt{m^*_{\mathrm{h}x}m^*_{\mathrm{h}y}}}}\right) + g_{\mathrm{e}}\sqrt{m^*_{\mathrm{e}x}m^*_{\mathrm{e}y}}\left(1 - e^{-\frac{2\pi n}{g_{\mathrm{e}}k_{\mathrm{B}}T\sqrt{m^*_{\mathrm{e}x}m^*_{\mathrm{e}y}}}}\right)\right] \tag{3}$$

Here, $m^*_{(\mathrm{h,e})(x,y)}$ are carrier effective masses, $g_{\mathrm{h}}$ and $g_{\mathrm{e}}$ are factors accounting for spin and valley degeneracy, $n$ is carrier concentration, $k_{\mathrm{B}}$ is Boltzmann constant, and $T$ is temperature. In a two-dimensional metal with parabolic bands, the screening wave number is independent of density at zero temperature. However, at finite $T$, screening is both density- and temperature-dependent, with increasing temperature leading to reduced screening; $T$=300 K is assumed here. The dependence of $s$ on temperature at several concentrations is shown for $m^* = 1$ in Supplementary Figure 1. $V_{\mathrm{eh}}\left(r\right)$ was obtained from $V_{\mathrm{eh}}\left(q\right)$ using Fourier transform. The excitonic binding energies $E_{\mathrm{b}}$ and wavefunctions were obtained by finding the eigenvalues and eigenfunctions of the effective mass Hamiltonian (Supplementary Equation 1). Gaussian basis set of size 6 was used to expand the ground-state excitonic wavefunction and calculate the matrix elements for the kinetic and potential energy of the Hamiltonian. The expansion was optimized using the downhill simplex algorithm.

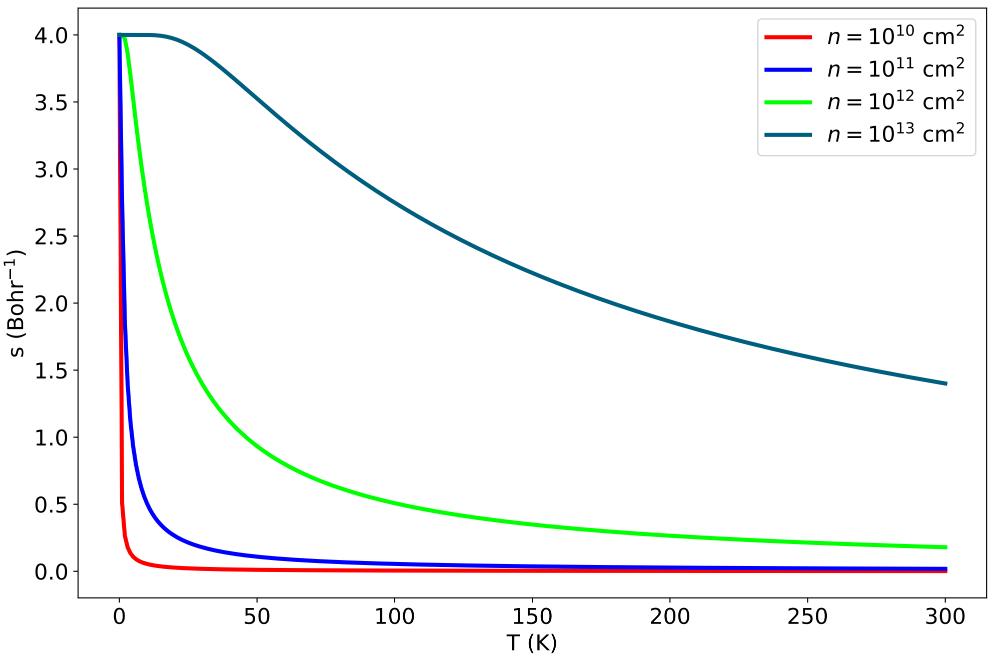


Supplementary Figure 1. Screening parameter $s$ in a metallic bilayer as a function of temperature and concentration, for $m^*_{(\mathrm{h,e})(x,y)} = m_\mathrm{e}$, $g_\mathrm{h} = 2$, $g_\mathrm{e} = 6$, as given by Supplementary Equation 3.

### 1.2 $Hf_2N_2I_2/Zr_2N_2Cl_2$ band overlap with SCAN functional

The band overlap is usually overestimated in calculations with LDA functional. We also used SCAN functional to get a more accurate estimate of the carrier density for $Hf_2N_2I_2/Zr_2N_2Cl_2$. The carrier density obtained with SCAN is $9 \times 10^{11}$ cm$^{-2}$, which as expected is lower than the value of $2 \times 10^{12}$ cm$^{-2}$ obtained with LDA functional. The effect of overestimating density with LDA is a change in the estimated critical temperature, with points in the semi-quantitative phase diagram Fig. 2b shifting to the left. Generally, the spread of $n$ values provided by different functionals would yield different estimates for $T_\mathrm{c}$. Our calculation with SCAN shows that the functional-dependent spread of $n$ values is smaller than the range of concentrations ($n \sim 10^{10} - 5 \times 10^{12}$ cm$^{-2}$) in which excitonic condensation should occur. In case of $Hf_2N_2I_2/Zr_2N_2Cl_2$, the estimated critical temperature would be 31 K or 14 K, depending on whether LDA or SCAN is used to estimate density. It is, however, clear that this system would remain within the dome for excitonic condensation.

## Supplementary Note 2

### 2.1 Tuning 2D materials to optimize excitonic instability

External factors such as strain, and possibly gating or changing the interlayer distance (yet not to weaken binding interaction), can fine-tune the carrier density, to access different regions of the phase diagram,

and increase the $T_c$. Supplementary Figure 2a shows the carrier density, in $Hf_2N_2I_2/Zr_2N_2Cl_2$ and $Sb_2Te_2Se$/BiTeCl, versus the external transverse electric field directed from donor to acceptor layer (left to right in Fig. 3); the dependence is linear as expected from the parallel plate capacitor model, yet the slope varies due to different screening in each case. It is seen that a field of ~1 V/nm can change the carrier density by $\sim 10^{12}$ cm$^{-2}$, raising the $T_c$ of material 3 to ~22 K, a quite noticeable shift in the Fig. 2b diagram. This also achieves crossover to the BKT regime. Similar perturbation can even be used to tune the band overlap in the hetero-bilayers lying on the flanks of Fig. 3, which were rendered above as suboptimal for excitonic condensation, if those are more readily synthesizable. For example, $Hf_2N_2I_2/SnS_2$ (see Supplementary Figure 4 for band structure) with a band overlap of ~0.2 eV has a carrier density of $\sim 1.4 \times 10^{13}$cm$^{-2}$, can be tuned by gating or the interlayer distance change (e.g. by inserting inert buffer layer of *h*-BN), as Supplementary Figure 2b shows. Approximately, more than 2 layers of *h*-BN when placed between the $Hf_2N_2I_2/SnS_2$ heterostructure can bring the carrier density to the range near $n < 5 \times 10^{12}$ cm$^{-2}$ optimal for excitonic condensation. Moreover, strain can be used to create band overlap in a small-gap hetero-bilayer with staggered bands: in $Sb_2Te_3$/BiTeI (see Supplementary Figure 6 for band structure), a negative band gap can be induced by in-plane tension, giving rise to free carriers, as seen in Supplementary Figure 2c. On the other hand, external electric field (gating) is routinely used to dope 2D materials and buffer *h*-BN layers are employed to either encapsulate 2D materials or create a multilayer heterostructure and superlattices [6].

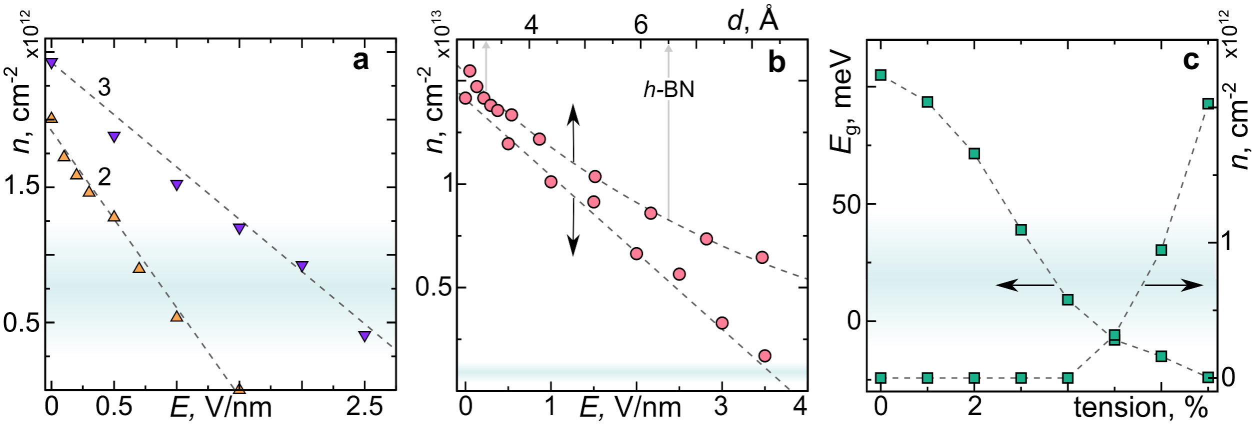


Supplementary Figure 2. Tuning 2D materials to optimize excitonic instability. (a) $n$ as a function of external electric field in (2) $Hf_2N_2I_2/Zr_2N_2Cl_2$ in (3) $Sb_2Te_2Se$/BiTeCl. (b) For $Hf_2N_2I_2/SnS_2$, $n$ as a function of field $E$ and interlayer distance (between inner chalcogens at equilibrium distance of 3.2 Å, and increased by ~3.3 Å if a *h*-BN layer were inserted). (c) For $Sb_2Te_3$/BiTeI, band gap and carrier density as a function of in-plane tension applied to both layers.

# Supplementary Note 3

## 3.1 Effect of SOC on band hybridization

We compare the bandstructures of $Sb_2Te_2Se$/BiTeCl, and $LiAlTe_2$/BiTeI calculated with and without spin-orbit coupling (SOC). In $Sb_2Te_2Se$/BiTeCl the bands hybridize due to SOC. There is a gap in the band structure calculated without SOC and including SOC splits the band, resulting in band crossing and hybridization (see Supplementary Figure 8). On the contrary in $LiAlTe_2$/BiTeI, a band crossing and hybridization is seen even without SOC (see Supplementary Figure 8). This hybridization can lead to interband tunneling processes and may fix the phase of the order parameter and destroy superfluidity [7]. However, the interband tunneling processes can be suppressed by using suitable dielectrics in between the two layers and superfluidity can be achieved.

## 3.2 Supercell structural stability

We performed supercell calculations of $Hf_2N_2I_2/SnS_2$ to confirm that van der Waals heterostructure is not prone to Peierls instabilities, thanks to weak electron-phonon interactions between the layers. In the $1 \times 1$ cell, the electron doped conduction band is at M point, while the hole doped valence band is at Γ point (Supplementary Figure 4). In the larger $2 \times 2$ cell the M point folds onto Γ point and, like $TiSe_2$, this material could undergo Peierls instability. To rule out this possibility we deformed the $2 \times 2$ cell by random $\sim \pm 0.2$ Å displacements of each atom. We found that the deformed $2 \times 2$ supercell reverts to the undeformed cell after ionic relaxation and does not show any structural distortions. Moreover, the $2 \times 2$ supercell bandstructure (Supplementary Figure 5) shows that there is no band gap opening and $Hf_2N_2I_2/SnS_2$ is still a semimetal. This affirms that $Hf_2N_2I_2/SnS_2$ and similarly other van der Waals heterostructures are not prone to Peierls instabilities, due to weak electron-phonon interactions between the layers. Moreover, collective excitations as performed in ref. [8] might be necessary to observe the spontaneous excitonic condensation mediated by purely electronic interaction in the hetrobilayers predicted in our study.

# Supplementary Note 4

## 4.1 Phase diagram for electrons and holes in a 2D bilayer

Excitons are composite bosons and display interesting phases. A phase diagram for electrons and holes in a 2D bilayer is shown in Supplementary Figure 9. In various regions, classical exciton gas (CEG), electron-hole plasma, Berezinskii-Kosterlitz-Thouless (BKT), Bardeen-Cooper-Schrieffer (BCS), and degenerate exciton Bose gas (DEBG) phases can exist. Due to reduced screening in 2D materials, individual excitons are stable at high temperatures and form a classical exciton gas (CEG) at low density ($n$) and high temperature. On decreasing the temperature, the CEG transitions to a degenerate exciton bose gas (DEBG) at temperature $k_B T_D = 2\pi\hbar^2 n/M$ [9]. Here, $M = m_e + m_h$. On decreasing the temperature further, the DEBG transitions to Berezinskii-Kosterlitz-Thouless (BKT) [10] phase at temperature $T_{BKT} = T_D/4$ . BKT is a two-dimensional analog of the three-dimensional BEC phase of excitons which, unlike BEC, lacks long-range phase coherence, but has local phase coherence and exhibits superfluidity. On increasing the exciton density at low temperature, the screening from carriers increases, which increases the exciton radius (decreases exciton binding energy) and individual excitons cannot be treated as point bosons. The BKT phase thus transitions to a BCS phase of excitons (analogous to that of Cooper pairs) [11]. BKT (BEC-like) and BCS are different

limits [12] of the same boson condensate state and may exhibit superfluidity. On increasing $n$ further at low $T$, the screening from other carriers is very high and it destroys the electron-hole binding and the BCS phase transitions to an electron-hole plasma state.

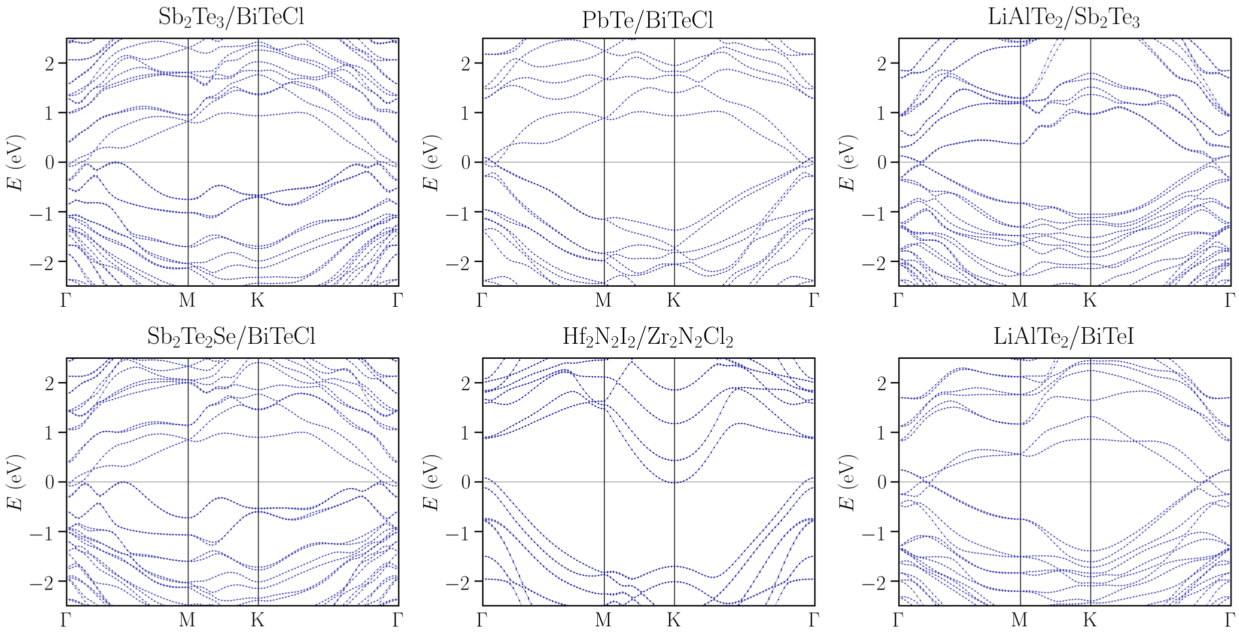


Supplementary Figure 3. Band structures of $Sb_2Te_3$/BiTeCl, PbTe/BiTeCl, $LiAlTe_2/Sb_2Te_3$, $Sb_2Te_2Se$/BiTeCl, $Hf_2N_2I_2/Zr_2N_2Cl_2$, and $LiAlTe_2$/BiTeI heterostructures.

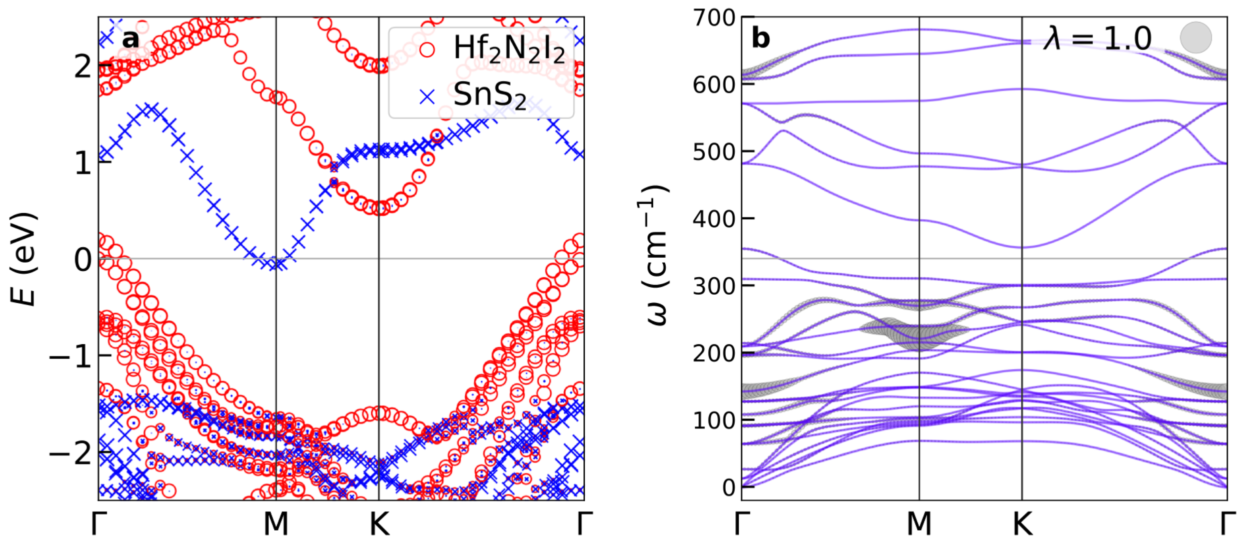


Supplementary Figure 4. (a) electronic band structure and (b) phonon dispersion of $Hf_2N_2I_2/SnS_2$ heterostructure.

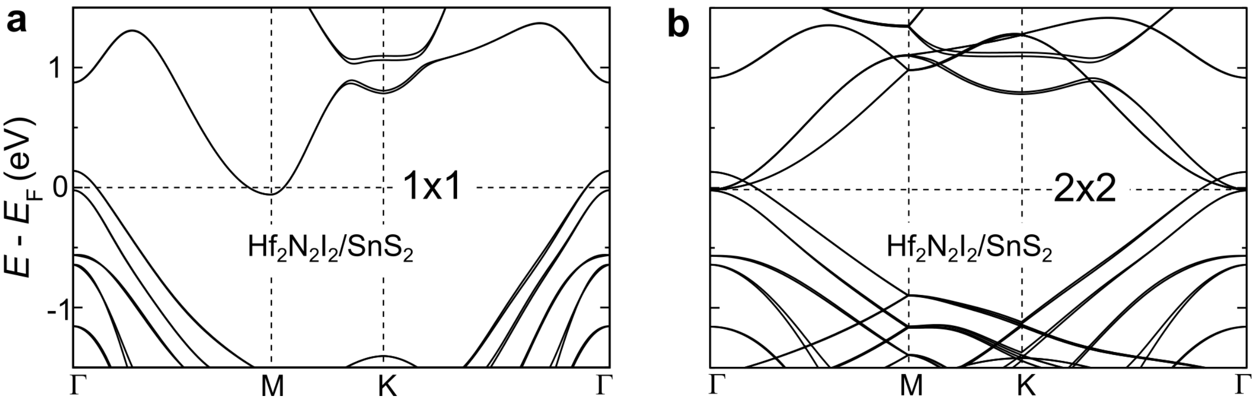


Supplementary Figure 5. Bandstructure of (a) $1 \times 1$ and (b) $2 \times 2$ supercell of $Hf_2N_2I_2/SnS_2$. The bands at M point in (a) are folded to the Γ point in (b). The $2 \times 2$ supercell bandstructure shows that $Hf_2N_2I_2/SnS_2$ is still a semimetal and is not prone to Peierls-like instabilities.

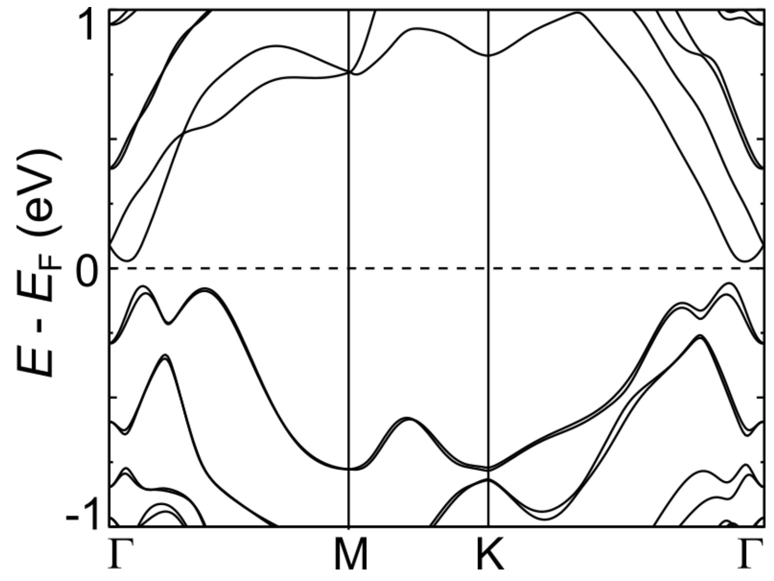


Supplementary Figure 6. Band structure of the staggered-gap $Sb_2Te_3$/BiTeI heterostructure.

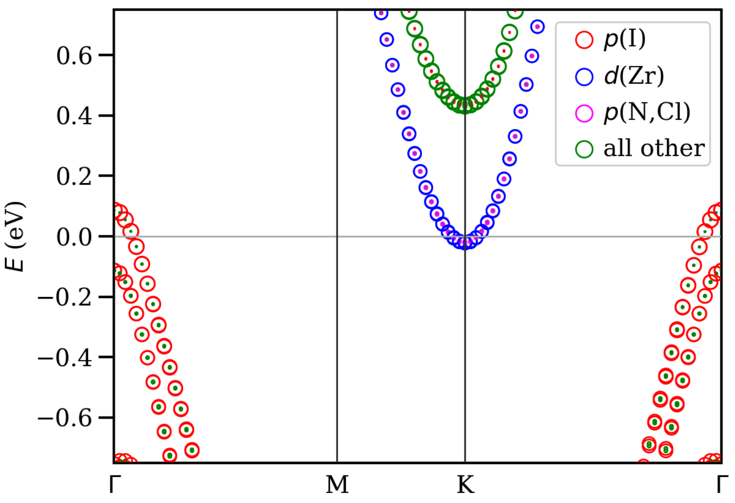


Supplementary Figure 7. Orbital projected band structure of $Hf_2N_2I_2/Zr_2N_2Cl_2$ .

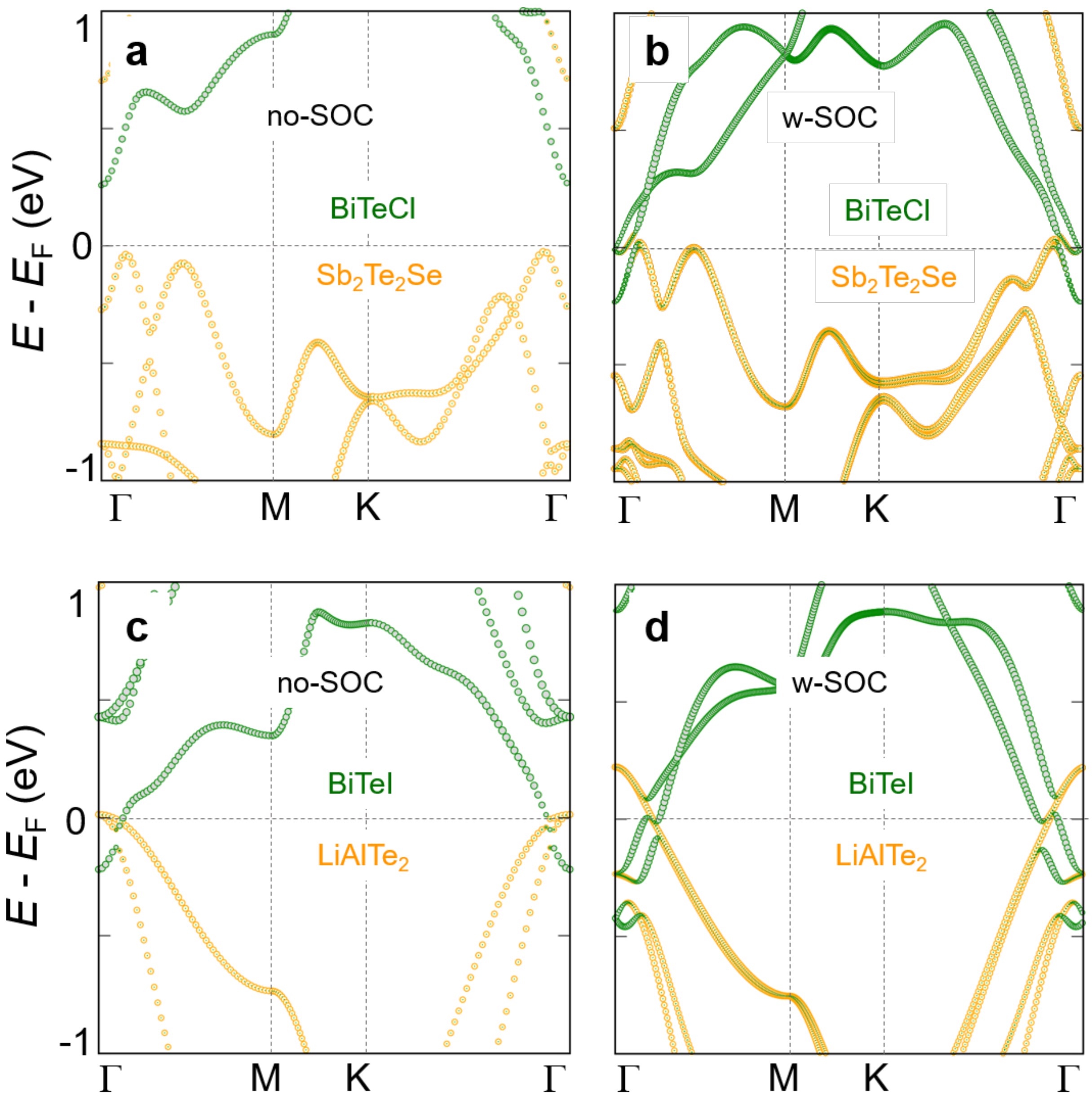


Supplementary Figure 8. Band structures of $Sb_2Te_2Se$/BiTeCl, and $LiAlTe_2$/BiTeI (a,c) without SOC and (b,d) with SOC.

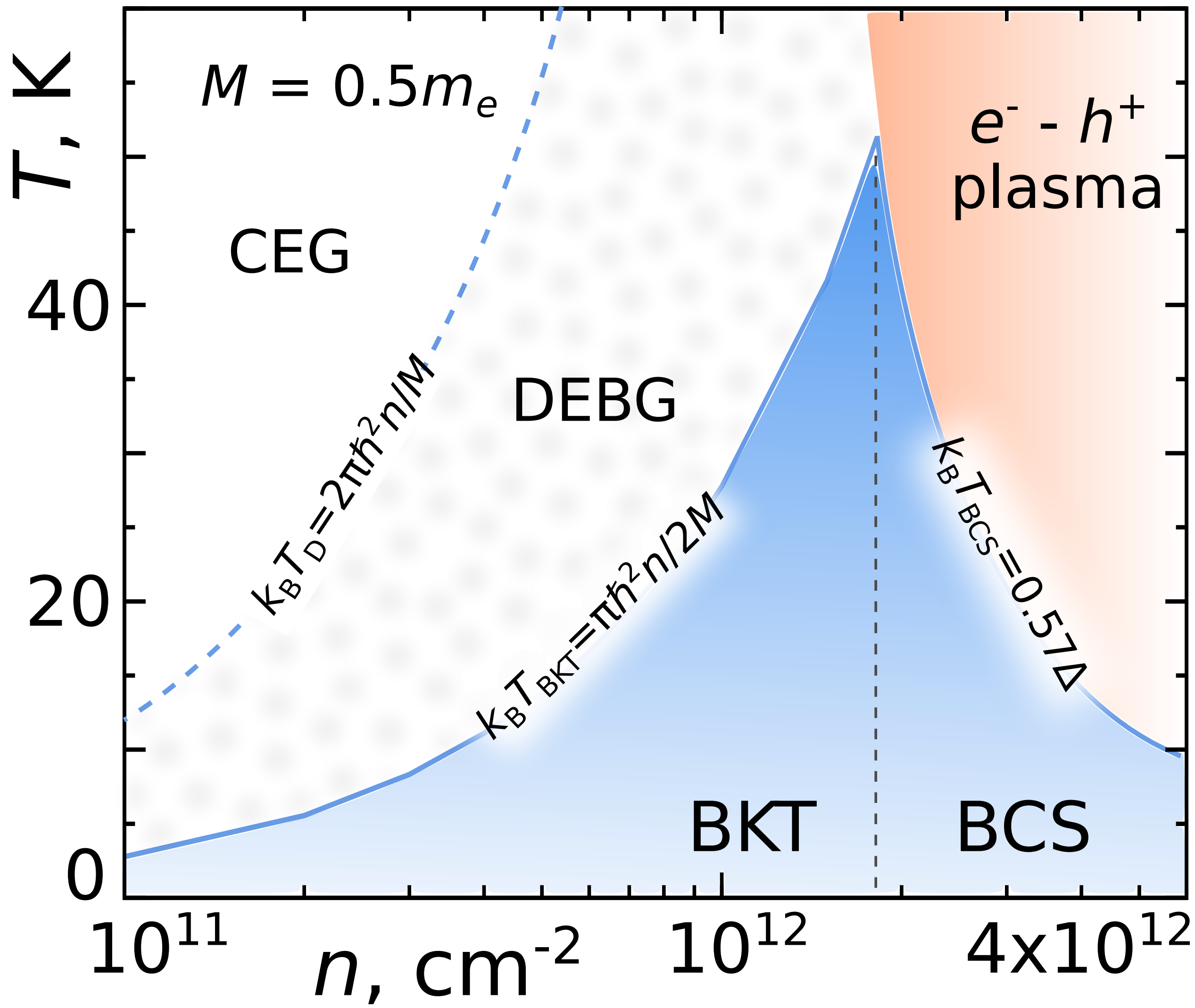


Supplementary Figure 9. Phase diagram for carriers in a 2D bilayer. Classical exciton gas (CEG), electron-hole plasma, Berezinskii-Kosterlitz-Thouless (BKT), Bardeen-Cooper-Schrieffer (BCS), and degenerate exciton Bose gas (DEBG) phases are shown.

| | $m^*_{\mathrm{h}x}$ | $m^*_{\mathrm{h}y}$ | $m^*_{\mathrm{e}x}$ | $m^*_{\mathrm{e}y}$ | $a\ \left(\mathring{A}\right)$ | $d\ \left(\mathring{A}\right)$ | $\varepsilon_{\mathrm{CBM}}$ (meV) |
|---|---|---|---|---|---|---|---|
| $Hf_2N_2I_2/SnS_2$ | -0.30 | -0.30 | 0.24 | 0.73 | 3.61 | 3.16 | -212 |
| $Sb_2Te_2Se/BiTeCl$ | -0.28 | -1.72 | 0.19 | 0.72 | 4.16 | 2.78 | -67 |
| $Hf_2N_2I_2/Zr_2N_2Cl_2$ | -0.30 | -0.30 | 0.60 | 0.60 | 3.61 | 3.73 | -47 |
| $LiAlTe_2/BiTeI$ | -0.07 | -0.35 | 0.14 | 2.09 | 4.28 | 2.79 | -38 |

Supplementary Table 1. Effective carrier masses $m^*$, lattice constants $a$, interlayer distances $d$, and positions of the CBM in $Hf_2N_2I_2/SnS_2$, $Sb_2Te_2Se/BiTeCl$, $Hf_2N_2I_2/Zr_2N_2Cl_2$, and $LiAlTe_2/BiTeI$ heterostructures. Effective mass values are at the VBM and CBM in principal axes in units of free electron mass $m_{\mathrm{e}}$. The position of the conduction band minimum, $\varepsilon_{\mathrm{CBM}}$, is given relative to the valence band maximum.

# Supplementary References


[1] Luqing Wang, Alex Kutana, and Boris I. Yakobson. Many-body and spin-orbit effects on direct-indirect band gap transition of strained monolayer $MoS_2$ and $WS_2$. *Annalen der Physik*, 526:L7–L12, 2014.

[2] Mitsuhiro Okada, Alex Kutana, Yusuke Kureishi, Yu Kobayashi, Yuika Saito, Tetsuki Saito, Kenji Watanabe, Takashi Taniguchi, Sunny Gupta, Yasumitsu Miyata, Boris I. Yakobson, Hisanori Shinohara, and Ryo Kitaura. Direct and indirect interlayer excitons in a van der waals heterostructure of hbn/ws2/mos2/hbn. *ACS Nano*, 12(3):2498–2505, 2018.

[3] Yu. E. Lozovik and V. I. Yudson. Feasibility of superfluidity of paired spatially separated electrons and holes; a new superconductivity mechanism. *JETP Lett.*, 22(11):274–276, 1975.

[4] Yu. E. Lozovik, S. L. Ogarkov, and A. A. Sokolik. Condensation of electron-hole pairs in a two-layer graphene system: Correlation effects. *Phys. Rev. B*, 86(4):045429, July 2012.

[5] Hartmut Haug and Stephan W Koch. *Quantum Theory of the Optical and Electronic Properties of Semiconductors*. World Scientific Publishing Company, 2004.

[6] S. J. Haigh, A. Gholinia, R. Jalil, S. Romani, L. Britnell, D. C. Elias, K. S. Novoselov, L. A. Ponomarenko, A. K. Geim, and R. Gorbachev. Cross-sectional imaging of individual layers and buried interfaces of graphene-based heterostructures and superlattices. *Nature Materials*, 11:764 EP –, Jul 2012.

[7] B. Zenker, H. Fehske, and H. Beck. Fate of the excitonic insulator in the presence of phonons. *Phys. Rev. B*, 90:195118, Nov 2014.

[8] Anshul Kogar, Melinda S. Rak, Sean Vig, Ali A. Husain, Felix Flicker, Young Il Joe, Luc Venema, Greg J. MacDougall, Tai C. Chiang, Eduardo Fradkin, Jasper van Wezel, and Peter Abbamonte. Signatures of exciton condensation in a transition metal dichalcogenide. *Science*, 358(6368):1314–1317, 2017.

[9] M. M. Fogler, L. V. Butov, and K. S. Novoselov. High-temperature superfluidity with indirect excitons in van der waals heterostructures. *Nature Communications*, 5(1):4555, Jul 2014.

[10] J M Kosterlitz and D J Thouless. Ordering, metastability and phase transitions in two-dimensional systems. *Journal of Physics C: Solid State Physics*, 6(7):1181–1203, apr 1973.

[11] Yu. E. Lozovik and V. I. Yudson. A new mechanism for superconductivity: pairing between spatially separated electrons and holes. *Zh. Eksp. Teor. Fiz.*, 71(3):738–753, 1976.

[12] P. Nozieres and S. Schmitt-Rink. Bose condensation in an attractive fermion gas: From weak to strong coupling superconductivity. *Journal of Low Temperature Physics*, 59(3):195–211, May 1985.
