# Supplementary material for: Heterobilayers of 2D materials as a platform for excitonic superfluidity
Source: Nat Commun. 2020 Jun 12;11:2989. doi: 10.1038/s41467-020-16737-0 (PMC7293212; doi:10.1038/s41467-020-16737-0)
Supplement: Supplementary file 2 — Description of Additional Supplementary Files [file 41467_2020_16737_MOESM2_ESM.docx]

**Description of Additional Supplementary Files**

**File name:** Dataset 1

**Description:** Optimized geometries for 351 heterostructures listed in Fig. 3.
